# Understanding the Effects of NaCl, NaBr and Their Mixtures on Silver Nanowire Nucleation and Growth in Terms of the Distribution of Electron Traps in Silver Halide Crystals

**DOI:** 10.3390/nano8030161

**Published:** 2018-03-14

**Authors:** Yunjun Rui, Weiliang Zhao, Dewei Zhu, Hengyu Wang, Guangliang Song, Mark T. Swihart, Neng Wan, Dawei Gu, Xiaobing Tang, Ying Yang, Tianyou Zhang

**Affiliations:** 1 Department of Applied Physics, Nanjing Tech University, Nanjing 210009, China; weiliangzhao100@126.com (W.Z.); wanghengyu@njtech.edu.cn (H.W.); dwgu@njtech.edu.cn (D.G.); xbingtang@126.com (X.T); yingyang@njtech.edu.cn (Y.Y.); tyzhang@njtech.edu.cn (T.Z.); 2 Department of Chemical and Biological Engineering, University at Buffalo (SUNY), Buffalo, New York, NY 14260, USA; deweizhu@buffalo.edu; 3 College of Chemistry and Molecular Engineering, Nanjing Tech University, Nanjing 210009, China; songguangliang75@sina.com; 4 School of Electronic Science and Engineering, Southeast University, Nanjing 210096, China; wn@seu.edu.cn

**Keywords:** silver nanowire, NaCl, NaBr, AgBr_1−*x*_Cl*_x_* crystal, electron trap distribution, critical size of multiply-twinned particles, transparent electrode

## Abstract

In recent years, many research groups have synthesized ultra-thin silver nanowires (AgNWs) with diameters below 30 nm by employing Cl^−^ and Br^−^ simultaneously in the polyol process. However, the yield of AgNWs in this method was low, due to the production of Ag nanoparticles (AgNPs) as an unwanted byproduct, especially in the case of high Br^−^ concentration. Here, we investigated the roles of Cl^−^ and Br^−^ in the preparation of AgNWs and then synthesized high aspect ratio (up to 2100) AgNWs in high yield (>85% AgNWs) using a Cl^−^ and Br^−^ co-mediated method. We found that multiply-twinned particles (MTPs) with different critical sizes were formed and grew into AgNWs, accompanied by a small and large amount of AgNPs for the NaCl and NaBr additives, respectively. For the first time, we propose that the growth of AgNWs of different diameters and yields can be understood based on the electron trap distribution (ETD) of the silver halide crystals. For the case of Cl^−^ and Br^−^ co-additives, a mixed silver halide crystal of AgBr_1−*x*_Cl*_x_* was formed, rather than the AgBr/AgCl mixture reported previously. In this type of crystal, the ETD is uniform, which is beneficial for the synthesis of AgNWs with small diameter (30~40 nm) and high aspect ratio. AgNW transparent electrodes were prepared in air by rod coating. A sheet resistance of 48 Ω/sq and transmittance of 95% at 550 nm were obtained without any post-treatment.

## 1. Introduction

Silver nanowires (AgNWs) have attracted much attention for use in devices like flexible touch screens, thin film solar cells, organic light emitting diodes and biomolecular sensors [1,2,3,4,5]. For use as transparent conductive electrodes, in place of the indium tin oxide (ITO) films that are widely used in current technologies, the AgNW electrode must exhibit high transmittance in the visible spectrum (>90% at 550 nm) and low sheet resistance (<50 Ω/sq) [4,5]. The properties of AgNW networks were reported to depend upon the aspect ratio, connectivity and overlap distribution of the AgNWs [6]. Use of AgNWs of high aspect ratio (defined as the ratio of length to diameter) improves the transmittance as well as the electrical conductivity. For thinner AgNWs, the absorbance associated with the localized surface plasmon resonance (LSPR) in the direction of the radius of the AgNWs is blue-shifted and the transmittance over a wide range of the visible spectrum is enhanced. At the same time, longer AgNWs are beneficial for achieving higher connectivity for a given overlap distribution, leading to lower sheet resistance. Therefore, thin AgNWs with high aspect ratio are desired for transparent conductive electrodes.

However, few successful examples of AgNWs with diameter below 30 nm have been reported using NaCl as an additive for the growth of AgNWs during the polyol process [7]. Typically, AgNWs larger than 70 nm were obtained with NaCl regardless of other variations in the synthesis methods [5,8,9,10,11,12]. When NaBr was used as the additive, the diameter was dramatically reduced to less than 30 nm but many silver nanoparticles (AgNPs) were observed in the product [13,14]. So, the yield of NWs using NaBr additive was lower than using NaCl. Moreover, some researchers have used both NaCl and NaBr additives to produce AgNWs with small diameters, some even approaching 20 nm [4,5,15,16,17]. However, many AgNPs were produced in these studies as well. The Cl^−^ and Br^−^ ions tend to form AgCl and AgBr crystals during the synthesis, respectively, which serve as heterogeneous nucleants for the growth of AgNWs. However, the evolution of AgCl and AgBr grains is quite different. Typically, AgBr-seeded AgNWs have a small diameter but are accompanied by many AgNPs. The large number of AgNPs (low yield of AgNWs) obtained by using NaBr additive is also related to the weak etching ability of Br^−^/O_2_ compared with that of Cl^−^/O_2_. In order to increase the percentage of NWs, other factors must be considered to precisely control the synthetic process, such as bubbled gas [12], injection rate of additive and reaction temperatures [14,16,17,18]. Tuning these complicated conditions to achieve high yield of AgNWs with small lateral size using Cl^−^ or/and Br^−^ mediated methods is still a great challenge.

Herein, we propose a facile method to synthesize AgNWs with diameter of 30~40 nm, aspect ratio up to 2100 and AgNW percentage yield above 85%, using Cl^−^ and Br^−^ co-additives under ambient atmospheric conditions. We found that AgNWs grew from multiply-twinned particles (MTPs) heterogeneously generated by electron trap (Ag_t_^+^) reduction on the surface of AgCl, AgBr or AgBr_1−*x*_Cl*_x_* grains. The dependence of the evolution of these silver halide crystals on the ETD and the free Ag^+^ concentration in solution are explored to understand the factors required to produce ultra-thin AgNWs in high yield. Finally, AgNWs with an aspect ratio of ~2100 were used to form a transparent conductive electrode with excellent optical and electrical performance.

## 2. Experimental

### 2.1. Synthesis of Silver Nanowires

All chemicals including silver nitrate (AgNO_3_, 99.8%), ethylene glycol (EG, 99.5%), sodium chloride (NaCl, 99.5%), sodium bromide (NaBr, 99.0%) and poly(vinyl pyrrolidone) (PVP, MW~58,000) were purchased from Shanghai Chemical Reagent Company and were used as received without further purification. All experiments were conducted under ambient conditions using the polyol method. In a standard synthesis, 0.25 g AgNO_3_ and 0.6 g PVP were separately dissolved in 5 mL EG, while 20 mL EG was added into a two-neck flask and heated at 160 °C for 5 min under magnetic stirring in an oil bath. Then 120 µL of NaCl solution in EG (300 mM) was pipetted into the flask, followed by the AgNO_3_ solution. After 1 min, the PVP solution was introduced and then the reflux pipe was fixed. After another 5 min, the stirring was stopped. This was taken as the starting time for AgNW growth (time = 0). Slight gray swirls were observed within 10 min indicating NW formation. The reaction was kept at 160 °C and continued for 60 min with the gray-white swirls in the mixture. In order to explore the role of Cl^−^, experiments were carried out with different concentrations of NaCl from 0.12 to 6 mM. In experiments using Br^−^ ions, the synthesis process was the same as the Cl^−^ case. Finally, the synthesis was carried out with mixed NaCl/NaBr additives to investigate the combined effects of Cl^−^ and Br^−^ ions. The detailed conditions of these experiments are summarized in Table S1. During the course of reaction, a small amount of mixture was sampled by pipette at different times. The products were washed with acetone and ethanol and centrifuged at 4000 rpm for 30 min and this was repeated three times. The final precipitates were stored in ethanol prior to microstructural and optical characterization.

### 2.2. Fabrication of Ag Nanowire Electrode

To make conductive films, microscope glass slides (Sail brand 7101, 25 × 75 × 1 mm) were thoroughly cleaned with detergent and washed with de-ionized water, sonicated in acetone, iso-propanol and ethanol each for 10 min and dried in an oven. The Ag nanowire suspension in ethanol was dropped onto the glass substrate. A glass rod (6 mm diameter) was then quickly moved over the AgNW solution by hand, spreading it across the glass into a thin, uniform film. The slides coated with AgNWs were dried in air for 20 min at room temperature.

### 2.3. Measurements

The crystal structures of the reaction products were investigated by X-ray diffraction (XRD) using a DX-2700 type diffractometer with Cu-Kα radiation (λ = 1.5406 Å). The morphology and microstructure of the samples were determined by scanning electron microscopy (SEM, JSM-5900, JEOL Ltd., Tokyo, Japan) and transmission electron microscopy (TEM, JEM 2010 UHR, JEOL Ltd., Tokyo, Japan). The distributions of diameter and length as well as the yield of AgNWs were obtained by analysis of the SEM images. The elemental analysis of the crystals was conducted by energy dispersive X-ray spectroscopy (EDX, NORAN System 7, Thermo Fisher Scientific Inc., Waltham, MA, USA). The ultraviolet-visible (UV-vis) absorbance spectra of the AgNW suspensions in ethanol and of conductive films on glass substrates were taken on a Perkin-Elmer Lambda 35 spectrometer. The sheet resistances of AgNW transparent films were measured using a four-point probe (ST2263, Suzhou Jingge Electronic Co. Ltd., Suzhou, China).

## 3. Results and Discussion

### 3.1. Characterization of AgNWs by SEM, XRD and Absorption Spectra

**Figure 1:**
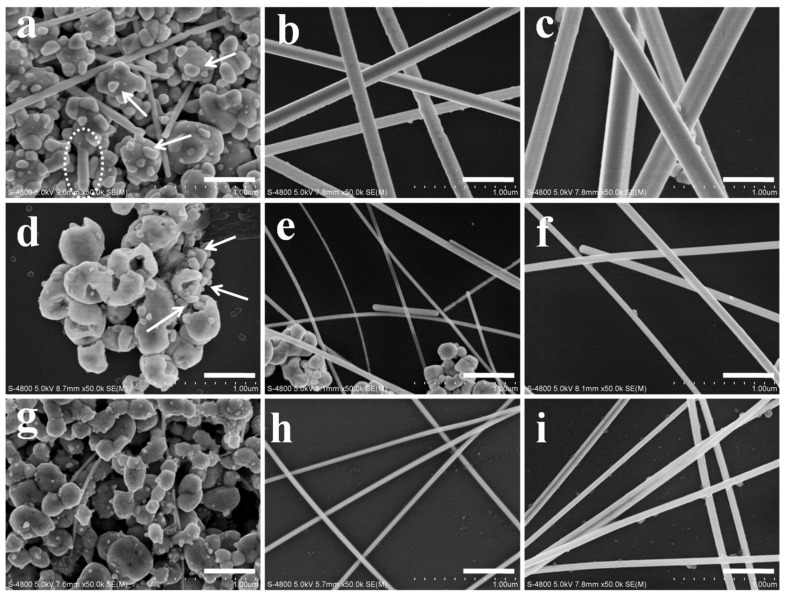
SEM images of AgNWs synthesized with different additives: NaCl (**a**–**c**), NaBr (**d**–**f**) and NaCl/NaBr (**g**–**i**) at three stages of 10 (left), 30 (middle) and 60 min (right column) in the polyol process. AgNPs were formed on the surface of AgCl crystals but inside the AgBr crystals, as indicated by arrows in (**a**,**d**), respectively. A fresh NW emanating from the AgCl surface was observed in (**a**), as marked by the dotted circle. The scale bar is 500 nm.

Figure 1 presents SEM images of the AgNWs synthesized with NaCl and NaBr additives. For the case of NaCl, at a short reaction time of 10 min, irregular, ellipsoidal (“potato-like”) AgCl crystals were decorated with AgNPs. Among these AgNPs, multiply-twinned particles (MTPs) must also exist because only MTPs can grow into AgNWs. Several thin AgNWs were observed in Figure 1a, which grew to diameters of 162 and 230 nm after growth times of 30 and 60 min, respectively. The same process was observed in the AgNW growth with NaBr additive. However, the diameter of AgNWs dramatically decreased to 27 nm and 80 nm for reaction times from 30 to 60 min, respectively. These AgNWs were much thinner than the AgNWs prepared using NaCl. In Figure 1a, MTPs (and AgNPs) were formed on the surface of AgCl crystals (indicated by arrows) and a fresh NW emanating from the AgCl surface was observed (dotted circle). However, MTPs (and AgNPs) were generated inside the “potato” shaped AgBr grains shown in Figure 1d. The position of MTPs (and AgNPs) was quite different for AgCl and AgBr grains. For comparison, Figure 1 also presents SEM images of AgNWs prepared with the NaCl/NaBr co-additives. We note that some AgNWs with very small diameter of ~25 nm were generated among the “potato-like” mixed silver halide crystals, as shown in Figure 1g. Increasing the growth time to 30 min, NWs with 40 nm diameter and 84 µm length were obtained. The parameters, such as diameter, aspect ratio and NW yield, of the AgNWs produced with these three different additives are tabulated in Table 1. The statistics of diameter and length of AgNWs are shown in Figure S1, illustrating that uniform AgNWs with small diameters and high aspect ratio were obtained for the NaCl/NaBr co-mediated sample. Figure S1 also demonstrates the swirls with different colors of densely opalescent, lightly gray and silver gray appearance when NaCl, NaBr and NaCl/NaBr, respectively, were used as additives.

**Figure 2:**
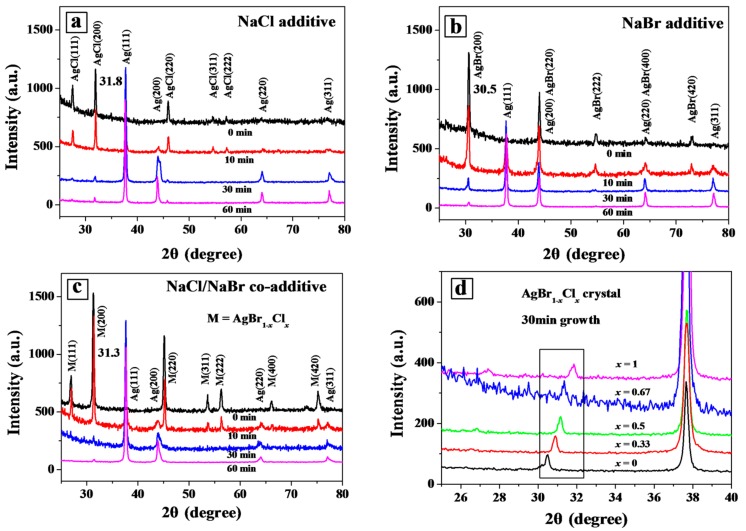
XRD patterns of samples obtained with NaCl (**a**), NaBr (**b**) and NaCl/NaBr (**c**) additives at different growth time. The diffraction peaks at 31.8°, 30.5° and 31.3° was observed from the AgCl, AgBr and AgBr_1−*x*_Cl*_x_* crystals, respectively. (**d**) Shifted diffraction peaks in AgBr_1−*x*_Cl*_x_* crystals synthesized with different molar ratio of *x* = [NaCl]/([NaCl]+[NaBr]).

To investigate the growth mechanism of AgNWs, XRD was carried out as shown in Figure 2. Two sets of diffraction peaks were observed in the AgNW sample prepared with NaCl additive. One was indexed to the (111), (200), (220), (311) and (222) planes of AgCl at 2θ = 27.0, 31.8, 45.5, 54.8 and 57.5, respectively. Another was indexed to the (111), (200), (220), (311) planes of Ag at 2θ = 37.8, 44.3, 66.4 and 77.8, respectively [19]. Figure 2a shows that diffraction peaks corresponding to AgCl gradually decreased, while the diffraction peaks of Ag increased correspondingly with increasing growth time. Weak diffraction from Ag, including the Ag(111) and Ag(200) peaks, could be observed for the 10 min sample. When the reaction time was increased to 30 min (or 60 min), additional peaks corresponding to Ag(220) and Ag(311) were increasingly obvious, while the AgCl related signals nearly disappeared. Similar evolution of AgNWs was observed when the NaCL additive was replaced with NaBr or NaCl/NaBr, as presented in Figure 2b,c. For the mixed additive, a mixed silver bromochloride AgBr_1−*x*_Cl*_x_* crystal was formed. The elemental content of the mixed AgBr_1−*x*_Cl*_x_* crystal was obtained from the EDX spectra (Figure S2). The existence of a single silver halide phase was evidenced by the diffraction peak at 2θ = 31.3°, differing from that of the individual AgCl and AgBr crystals or an AgCl/AgBr mixture (Figure S3a). This diffraction position of 31.3° was invariant with growth time as shown in Figure 2c. In addition, the AgBr_1−*x*_Cl*_x_* crystal with different molar ratio *x* (defined as *x* = [*NaCl*]/([*NaCl*]+[*NaBr*]) in the precursor solution for simplicity) could be synthesized and the diffraction peaks were shifted to higher angles with increasing *x,* as shown by XRD patterns in Figure 2d [20]. Similar to the mixed AgBr_1−*x*_Cl*_x_* (*x* = 0.67) crystal, the diffraction positions (31.1° and 30.8°) of AgBr_1−*x*_Cl*_x_* crystal (*x* = 0.5 and 0.33) were also unchanged during the AgNW growth process, which indicated that the composition of the AgBr_1−*x*_Cl*_x_* grains was independent of the growth time, as shown in Figure S3b,c.

**Figure 3:**
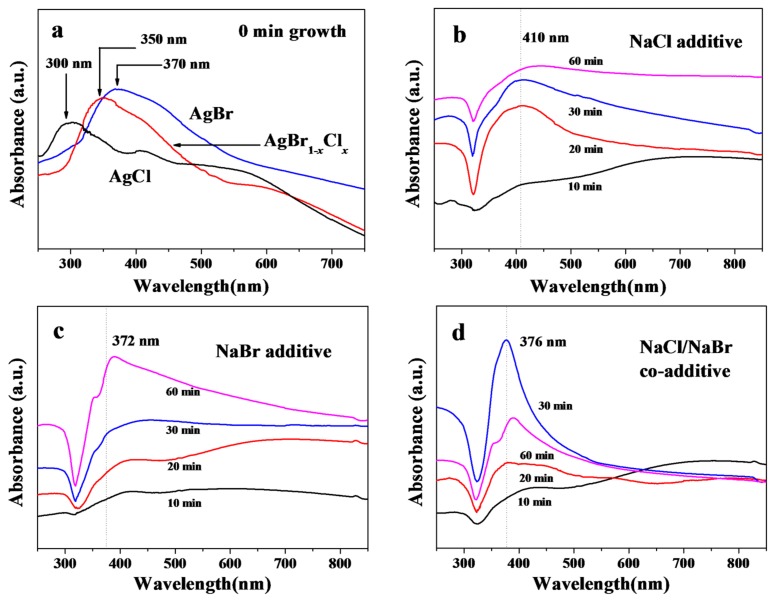
UV-vis absorbance spectra (**a**) of AgCl, AgBr and AgBr_1−*x*_Cl*_x_* crystals obtained at the beginning of synthesis and of the AgNW samples obtained with NaCl (**b**), NaBr (**c**) and NaCl/NaBr (**d**) additives. The vertical dotted lines in (**b**–**d**) indicate the positions of LSPR for the 30 min growth samples.

We also used UV-vis absorbance to follow the growth evolution of the AgNWs. Figure 3a shows the absorbance spectra of products at the beginning of the reaction (0 min). The peaks at 300, 350 and 370 nm originate from AgCl, AgBr_1−*x*_Cl*_x_* and AgBr crystals for the NaCl, NaCl/NaBr and NaBr additives, respectively [19,21], consistent with the SEM images of Figure 1 and XRD patterns of Figure 2. After a growth time of 10 min, the absorbance at 420 nm and in the red range (>600 nm) was enhanced because of the generation of the small AgNPs and MTPs [22,23]. Subsequently, under the influence of PVP, MTPs grew into long AgNWs. The diameter of AgNWs also increased, as reflected by the red-shift of the LSPR of the AgNW sample in Figure 3 [12,17,24]. The LSPR position is higher for AgNWs prepared with the NaCl additive than for the NaBr counterpart. In Figure 3b,c, 410 and 372 nm absorption bands were observed for the NaCl and NaBr additive samples at 30 min growth time, respectively, which correspond to AgNWs of 162 and 27 nm diameters as shown in SEM images. Moreover, the full width at half maximum (FWHM) of the AgNW LSPR broadened during the growth process owing to the broad diameter distribution of AgNWs prepared with NaCl additive (Figure 3b) or the broad size distribution of NPs produced in the presence of NaBr (Figure 3c), consistent with prior reports [11,12]. However, the FWHM was greatly reduced for samples produced with the mixed NaCl/NaBr additive. In Figure 3d, the absorption band with a maximum at 376 nm and with narrow FWHM (70 nm) was observed for the 30 min growth sample, which reflects the uniform diameter (~40 nm) of the AgNWs in this sample.

### 3.2. Analysis of AgNW Growth Mechanism

Clearly, AgNWs grew from the AgCl, AgBr, or AgBr_1−*x*_Cl*_x_* crystal grains in our experiments, as previously reported [25,26,27,28] and the silver halide identity strongly influenced the final morphology of the AgNWs (including diameter, length and yield), as shown in Figure 1. In recent years, some researchers reported that the diameter of AgNWs could be determined by the size of AgCl or AgBr crystal formed in the initial step of the polyol synthesis. Wang et al. found that the diameter of AgNWs could be well controlled by tailoring the size of silver halide seeds. Different sizes of AgBr (110–180 nm) and AgCl (115–300 nm) grains led to the formation of AgNWs with 51–53 nm and 63–68 nm diameter, respectively [29]. Moreover, when the NaCl and NaBr were both applied in the polyol process, the size of silver halide nanoparticles further decreased to 30 nm. As a result, ultrathin AgNWs with diameter of 20 nm and high aspect ratio of 2000 could be achieved [5]. However, in our case, although AgNWs with significantly different diameter (27 and 162 nm for NaBr and NaCl additive, respectively) were observed, we do not attribute this variation to the sizes of AgBr and AgCl crystals. They both have the similar sizes of 300–500 nm as shown in Figure 1.

On the other hand, the concentration of additives could also affect the diameter of AgNWs [5,13,25,26,27,29]. Figure S4 presents the SEM images of AgNWs synthesized with NaCl additive at different concentrations. With increasing NaCl concentration from 0.12 to 6.0 mM, the mean diameter of the AgNWs decreased from 162 to 135 nm (Δ*d* = 27 nm) for the 30 min growth samples. AgCl crystals can not only act as heterogeneous nucleation centers for the growth of AgNWs but also reduce the free Ag^+^ concentration. This is similar to the low injection rate of precursor of AgNO_3_ sometimes employed in polyol synthesis to reduce AgNW diameter [14,18]. The same trend was observed using the NaBr additive. AgNWs with 30 and 27 nm (Δ*d* = 3 nm) were obtained for NaBr concentrations of 0.12 and 1.2 mM, respectively, as presented in the SEM images of Figure S5. When the NaBr concentration was further increased to 6.0 mM, AgNWs did not form. Clearly, the change in NW diameter (Δ*d*) due to changes in additive concentration is much smaller than that resulting from changing the identity of the silver halide additive (Figure 1).

**Figure 4:**
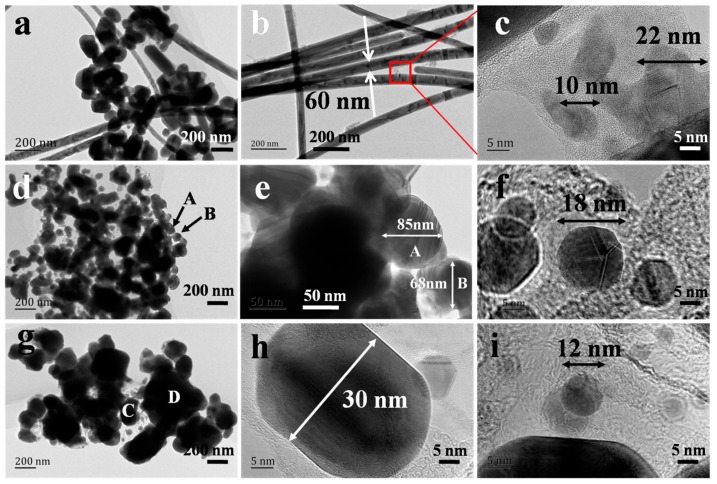
TEM images of AgNW samples for 10 min growth time with NaCl (**a**–**c**), NaBr (**d**–**f**) and NaCl/NaBr (**g**–**i**) additives.

The diameter of AgNWs was reported to be strongly influenced by the critical size of MTPs [17,26,30]. Schuette and Buhro studied the growth mechanism of AgNWs and identified a critical size of MTPs, beyond which lengthening starts. They found the mean diameter near the onset of growth in the length dimension to be 20 to 30 nm and the final diameter to be 2.5 times the critical size. For the NaBr additive, AgNWs with diameter below 50 nm were obtained [13]. This implies that the critical size of MTPs heterogeneously nucleated on the surface of AgBr crystals was below 20 nm, if the same proportion between the critical size and the final diameter of AgNWs is assumed for AgNWs grown from AgCl and AgBr. To explore the critical size of MTPs for the growth of AgNWs in our case, TEM images of AgNW samples (10 min growth time) were obtained, as shown in Figure 4. At this stage, AgNWs were observed for the NaCl additive case with a uniform diameter of 60 nm, as shown in Figure 4b. Some MTPs with sizes of 22 or 10 nm were generated. Therefore, the critical size of MTPs was in the range of 22~60 nm. For the case of NaBr additive, only AgNPs exist, among which one MTP with a size of 18 nm was observed, as shown in Figure 4f. This small MTP offers the possibility for the growth of ultra-thin AgNWs with uniform diameter of 27 nm (Figure 1e). So, the critical size of MTPs was 18~27 nm for the case of AgBr crystal. MTPs with critical size smaller than 30 nm were formed in the presence of AgBr_1−*x*_Cl*_x_* crystal, as shown in Figure 4h. Thus, we infer that when the lateral growth of AgNWs was constrained, the production of 40, sub-20 and sub-30 nm thin AgNWs were possible for the NaCl, NaBr and NaCl/NaBr additives, respectively [7,14,17]. However, in many cases, the growth of AgNWs occurs in both lateral and longitudinal directions.

The origin of differences in MTP critical size in the presence of AgCl and AgBr has not been well-understood. AgCl and AgBr crystals are prominent in photographic processes due to their photosensitivity. Both AgCl and AgBr exhibit an indirect band gap, which results in a long lifetime of the excited state before recombination of a photoelectron and a hole [31]. This increases the probability of combination of a photoelectron with interstitial silver, Ag*_i_*^+^, existing in AgCl and AgBr crystals. As a result, a latent image is formed. However, latent image formation is faster in AgBr than that in AgCl grains, partially due to the different effective mass of electrons (0.215 *m*_0_ vs. 0.302 *m*_0_) and Hall mobility (60 vs. 50 cm^2^ V^−1^ s^−1^) for AgBr and AgCl, respectively [32]. In addition, Tani et al. [33,34] studied electronic properties and photographic behavior of AgCl and AgBr crystals and reported that the concentration of interstitial Ag*_i_*^+^ within AgBr grains is two orders of magnitude larger than in AgCl grains, while on the AgBr grain surface, the number of Ag*_k_*^+^ at kink sites is much lower than on the AgCl crystal surface. The interstitial Ag*_i_*^+^ within the grains and the Ag*_k_*^+^ at the kink sites on the grain surface could both act as electron traps for latent image formation. Here, we define the electron traps, together, as Ag*_t_*^+^. Therefore, Ag*_t_*^+^ is present mainly in the interior of AgBr grains but mainly on the surface of AgCl grains.

### 3.3. Our Proposed ETD Mechanism for the Growth of AgNWs

**Figure 5:**
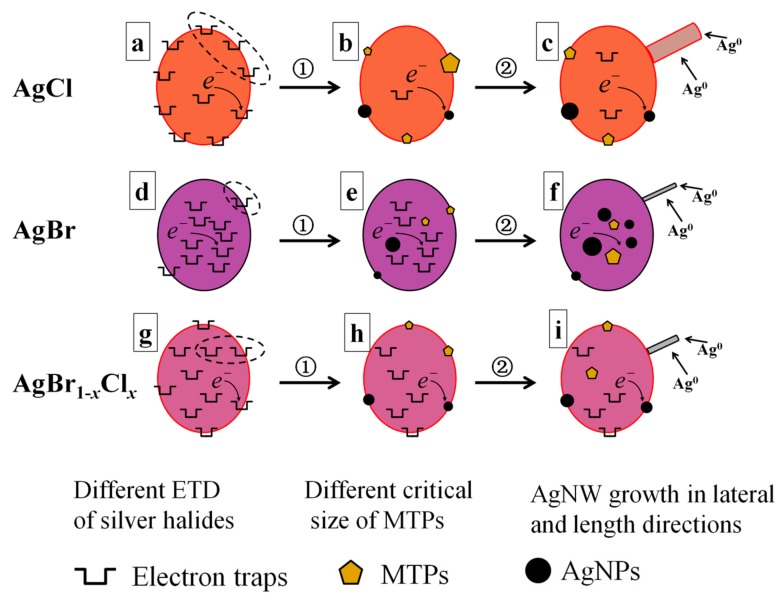
Schematic illustration of the growth of AgNWs undergoing two steps (①,②) for the three types of silver halides of AgCl (**a**–**c**), AgBr (**d**–**f**) and AgBr_1−*x*_Cl*_x_* (**g**–**i**) crystals.

Based on the quite different electron trap distributions (ETD), herein we propose a mechanism that explains the difference in growth of AgNWs from the different silver halide crystals, as schematically illustrated in Figure 5. The growth of AgNWs occurs in two steps: (1) Formation of MTPs of critical size; and (2) AgNW growth in both lateral and length directions. The fact that the electron traps (Ag_t_^+^) in the silver halides could be reduced to Ag atoms (Ag^0^) by EG during the polyol process is generally accepted. These Ag atoms can agglomerate to form Ag seeds fluctuating between MTPs and single-crystal seeds, as described in the middle column of Figure 5. The former (thermodynamically stable) would grow into AgNWs, while the latter (kinetically stable) would grow into Ag nanoparticles (AgNPs) [30]. Once the MTPs reach the critical size, they rapidly grow in length to produce AgNWs. Notably, only a single NW was preferentially emanated from one crystal of silver halide in our studies and in Ref. [25].

Note that ETD would affect not only the critical sizes of MTPs but also the yield of AgNWs for the case of NaCl and NaBr additives. As shown in Figure 5a,d, electron traps (Ag_t_^+^) are mainly located on the surface of AgCl grains but mainly in the interior of AgBr grains. So, on the AgCl surface, the MTPs heterogeneously generated are apt to grow to a relatively large size because the number of reduced Ag^0^ in the vicinity is high, as labeled by the dashed oval in Figure 5a. On the contrary, relatively few Ag^0^ are present on the AgBr surface (labeled by the dashed oval in Figure 5d) and would aggregate into a small sized MTP. Compared with the AgCl crystal, the concentration of Ag*_t_*^+^ inside the AgBr crystal is higher. Thus, many MTPs and AgNPs are formed in the interior of the AgBr grains (Figure 5e). They became larger due to Ostward ripening but could not grow into AgNWs due to the absence of PVP inside the AgBr grains. Only the MTPs formed on the surface of AgBr grains would grow into AgNWs after their sizes exceed the critical value. Therefore, more NPs were generated when the NaBr additive was employed, as illustrated in Figure 5f. The yield of AgNWs was as low as 30%, as shown in Figure 1.

In fact, the growth of MTPs and NPs inside the AgBr crystal can burst the grains of AgBr. So many small AgBr crystals were produced. Figure 4e shows a TEM image of AgNWs grown with NaBr additive for 10 min growth time. Some 30~100 nm AgBr grains were observed (labeled with A and B), whose chemical composition was confirmed by EDX, as shown in Figure S6. Similar to the report of Wang et al., we believe that such small sized AgBr crystals are beneficial to generate MTPs with small critical size for the growth of ultra-thin AgNWs when NaBr additive was employed in the polyol synthesis [29].

After reaching the critical size illustrated in the middle column of Figure 5, the MTPs will grow into AgNWs in the both lateral and longitudinal directions. The final diameter and length of AgNWs was also dependent on several factors, such as the PVP concentration, temperature and amount of Ag^+^ precursor [12,18]. In our experiment, only the type of silver halide changed. The solubility product (*K_sp_* = 1.6 × 10^−10^) of AgCl is larger than that of AgBr (*K_sp_* = 4.9 × 10^−13^), which results in higher delivery rate as well as higher free Ag^+^ concentration in the solution [35]. On the other hand, the concentration of free Ag^+^ ions will also increase because Cl^−^/O_2_ can act as an etchant to dissolve AgNPs [36,37,38]. The etching ability of Cl^−^/O_2_ is stronger than that of Br^−^/O_2_. The free Ag^+^ ions are the primary source of NW growth [25]. Therefore, the growth of AgNWs (in both diameter and length) for the NaCl case was much faster than that for NaBr, as shown in Table 1. In detail, with the increasing of growth time from 10 to 30 min, the diameter of AgNWs was enlarged from 60 to 162 nm (2.7 times) and the aspect ratio reached 360. For the case of NaBr additive, 18 nm MTPs were formed (Figure 4f), which would grow into AgNWs with diameter of 27 nm (1.5 times) and aspect ratio of 230 at the growth time of 30 min, as shown in Figure 1.

With NaCl/NaBr co-additives, AgNWs with small diameter, high aspect ratio (up to 2100) and high yield were obtained. Although many researchers have studied how mixed AgBr_1−*x*_Cl*_x_* crystal affect photographic processes, such as latent image formation [39,40,41,42], the effect on AgNW growth of AgBr_1−*x*_Cl*_x_* grains in the polyol synthesis has not been reported. If we consider the different ETD characteristics of AgCl and AgBr crystals, we can expect that the ETD for AgBr_1−*x*_Cl*_x_* crystals would be uniform for some value of *x*. Figure 5g indicates the electron traps (Ag*_t_*^+^) existing uniformly both on the surface and within the AgBr_1−*x*_Cl*_x_* grains. This uniform ETD promotes the formation of MTPs of intermediate critical size and a high yield of AgNWs. In Figure 4h, a short nano-rod with diameter of 30 nm and length of 0.04 µm was formed. Based on our analysis above, the dissolution rate of AgBr_1−*x*_Cl*_x_* and the etching ability of Cl^−^/Br^−^/O_2_ for the NaCl/NaBr co-additive case are moderate compared with that of individual NaCl or NaBr additive case. So, the free Ag^+^ ions in the solution were exactly suitable for the AgNW growth. As shown in Figure 1, AgNWs with 40 nm diameter and 84 µm length (aspect ratio = 2100) were obtained when the growth time was 30 min for the AgBr_1−*x*_Cl*_x_* crystal sample. In this case, the yield of AgNWs was as high as 90%.

### 3.4. AgNW Diameter Controlled by NaCl/NaBr Concentration

With the uniform ETD, AgBr_1−*x*_Cl*_x_* is beneficial for the formation of MTPs with small critical size and AgNWs with excellent yield and high aspect ratio. However, the morphologies (proportion of AgNWs and AgNPs) were dependent on the molar ratio of Cl/Br for the case of NaCl and NaBr co-additive [5,15,17]. Similar to the AgBr crystal, electron traps of Ag*_t_*^+^ were mainly located inside of the crystal of AgBr_1−*x*_Cl*_x_* if the ratio of Cl/Br was low. Figure S7 presents the morphologies of products obtained with Cl/Br molar ratio of 1. Many NPs were observed due to nucleation of NPs and MTPs within the crystal, which could not grow into AgNWs. The diameter was very small (25 nm) but the yield of NW was as low as 20%. On the other hand, larger Cl/Br molar ratio (>2) will result in the formation of thick AgNWs [5]. Therefore, the Cl/Br molar ratio is a very important factor in the growth of NW.

**Figure 6:**
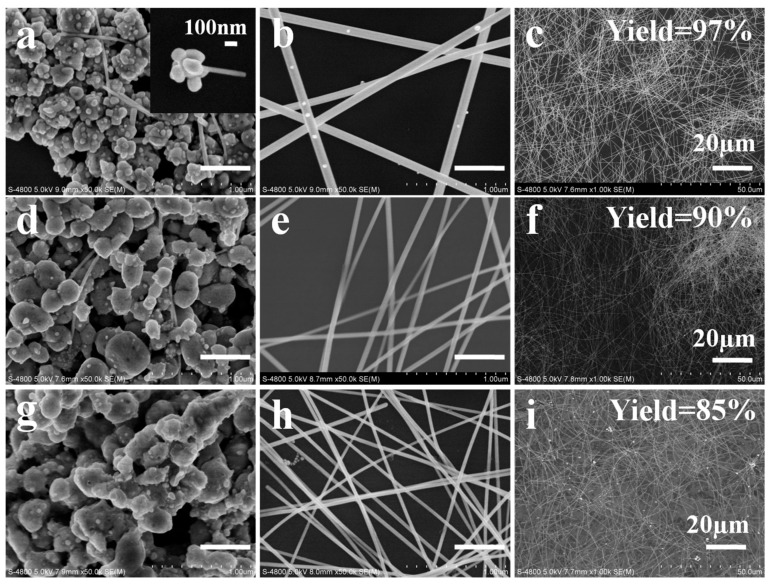
SEM images of AgNWs obtained with NaCl/NaBr co-additive of different concentration of 0.12/0.06 (**a**–**c**), 1.2/0.6 (**d**–**f**) and 6.0/3.0 mM (**g**–**i**) at growth times of 10 (left column) and 30 min (middle column), respectively. The scale bar is 500 nm. Low magnification SEM images of AgNWs at 30 min growth time are shown in the right column.

We obtained small diameter (40 nm) AgNWs with high aspect ratio of 2100 using a 2:1 ratio of NaCl/NaBr (1.2/0.6 mM). The diameter further decreased when the concentration of NaCl/NaBr increased, which is related to the decreased concentration of free Ag^+^ ions in the solution after the formation of AgBr_1−*x*_Cl*_x_*. Figure 6 shows SEM images of AgNWs obtained with different concentrations of NaCl/NaBr, while keeping the molar ratio of Cl/Br fixed at 2. The parameters of these AgNWs were also listed in Table 1. For the samples with low NaCl/NaBr concentration of 0.12/0.06 mM, several AgNWs with small diameter of 20~25 nm were observed on the surface of AgBr_1−*x*_Cl*_x_* crystal when the growth time was 10 min, as shown in Figure 6a. This value of diameter (20~25 nm) is in agreement with the critical size of MTPs for AgBr_1−*x*_Cl*_x_* crystals discussed above. However, the initially ultra-thin AgNWs rapidly grow into thick ones (94 nm) for the 30 min growth time because of the high concentration of free Ag^+^ ions in the solution. With increasing concentration of NaCl/NaBr, the concentration of free Ag^+^ ions in the solution decreases. As a result, 40 and 29 nm AgNWs were obtained for the cases with NaCl/NaBr concentrations of 1.2/0.6 and 6.0/3.0 mM, respectively, as shown in Figure 6e,h.

The decreased diameter of AgNWs was characterized by UV-vis absorbance spectroscopy, as shown in Figure S8a. With increasing concentration of NaCl/NaBr additives, the LSPR peaks are blue-shifted, which shows the decreasing diameter of AgNWs. In addition, the FWHM of the absorbance peak was reduced, which indicates that more uniform AgNWs with small diameter were formed. The standard deviation in diameter of 3.8 nm for the high co-additive concentration sample is much smaller than that of 6.9 (11.5) nm for the medium (low) co-additive concentration sample, as shown in Figure S1 and S9. On the other hand, a high concentration of NaCl/NaBr also leads to high aspect ratio AgNWs, as characterized by SEM images as well as by the Ag(111)/Ag(200) intensity ratio, as shown in Figure S8b. For the samples with 1.2/0.6 and 6.0/3.0 mM NaCl/NaBr additives, the diffraction intensity ratio of Ag(111)/Ag(200) was increased to 8.8 and 7.0, respectively, much larger than that of the sample synthesized with individual NaCl or NaBr additives. Therefore, we conclude that high concentration of NaCl/NaBr will be helpful for producing AgNWs with small diameter and high aspect ratio. However, when the concentration of NaCl/NaBr was high, some AgBr_1−*x*_Cl*_x_* crystals initially formed could not be dissolved completely. The peaks corresponding to residual AgBr_1−*x*_Cl*_x_* crystals in the XRD pattern were labeled with the arrows in Figure S8b. So, the yield of NW is decreased to 85% with the increase of NaCl/NaBr concentration to 6.0/3.0 mM, as shown in Figure 6.

Many research groups have chosen a Cl/Br of 2 to synthesize the ultra-thin AgNWs, as summarized in Table S2. For this ratio of NaCl/NaBr additive, the obtained AgNWs have both small diameter (<30 nm) and high aspect ratio (>1000). Although the diameter can be adjusted by the concentration of co-additives, the yield of AgNWs will also be changed. Our result of AgNWs (40 nm diameter, 2100 aspect ratio and 90% yield of AgNWs) is appealing compared with these similar studies in literature [4,5,14,15,16,17].

### 3.5. Properties of AgNW Transparent Electrodes

**Figure 7:**
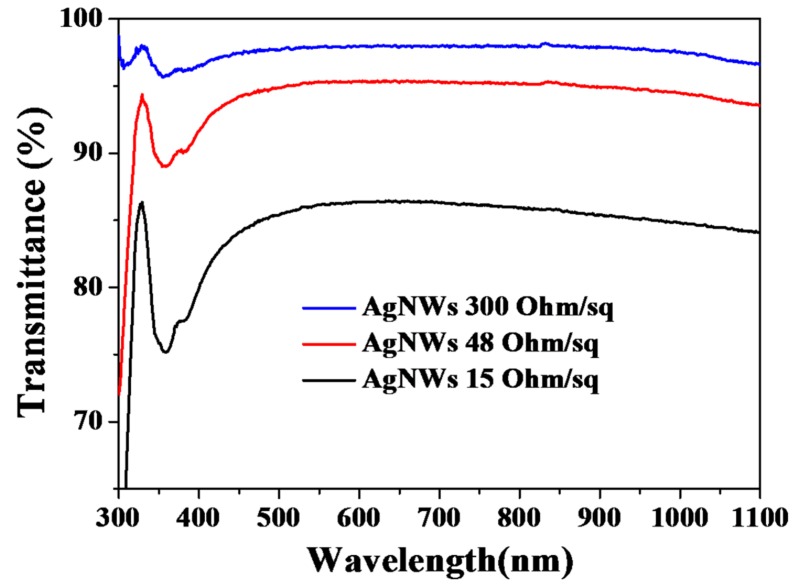
Optical transmittance of AgNW films, after subtraction of substrate absorbance.

Finally, we used the AgNWs with highest aspect ratio (2100 for Sample C_2_) to fabricate electrodes on glass substrates at room temperature by glass rod coating. Figure 7 shows the transmittance of AgNW films (excluding substrate absorbance) with various sheet resistances. The transmittance decreased with increasing electrical conductivity. At 550 nm, the transmittance reached 98, 95 and 86% when the sheet resistance was 300, 48 and 15 Ω/sq, respectively. This is comparable to the work of other researchers [4,5,15]. Digital photographs (Figure S10) and optical microscope images (Figure S11) of these three AgNW films on the glass substrates show that different densities of AgNWs lead to different transparency and sheet resistance. Thanks to the thin (40 nm) and long (84 µm) AgNWs, a conductive network could be formed with low density of AgNWs, which results in the high transmittance (95%) and low sheet resistance (48 Ω/sq). On the other hand, the electrical properties of the AgNW films could be improved by welding the junctions between AgNWs via various post treatments, such as thermal annealing, mechanical pressing and salt treatment [4,5,15,35,43,44,45]. This work is under way in our laboratory.

## 4. Conclusions

We have systematically investigated the roles of NaCl and NaBr additives in the polyol synthesis of AgNWs under ambient atmosphere. During the process, AgCl or AgBr crystals were formed as seeds for the growth of AgNWs. However, the evolution of AgCl and AgBr grains were quite different. Electron traps, Ag*_t_*^+^, were mainly located on the surface of AgCl crystals but inside the AgBr crystals. This electron trap distribution (ETD) influences the critical size of MTPs and the yield of AgNWs. As a result, 162 vs. 27 nm in diameter and 94 vs. 30% yield of AgNWs were obtained for the NaCl and NaBr additive samples, respectively. Based on the different ETD of silver halides, a growth mechanism for AgNWs of different aspect ratio was proposed. Moreover, for the first time, we clarified the behavior in the presence of mixed silver halide crystals of AgBr_1−*x*_Cl*_x_*, which formed when NaCl and NaBr were employed together. The AgBr_1−*x*_Cl*_x_* crystal has a uniform ETD, resulting in MTPs with intermediate critical size as well as a moderate concentration of free Ag^+^ ions in the solution. Therefore, thin (30~40 nm) and long (aspect ratio up to 2100) AgNWs were obtained. The diameter could also be controlled by varying the concentration of NaCl/NaBr additive because of the variable concentration of free Ag^+^ remaining in the solution after the formation of AgBr_1−*x*_Cl*_x_* crystals was completed. Although AgNWs with small diameter (30~40 nm) and high aspect ratio (up to 2100) in high NW purity (85% yield) were obtained in our experiment, further studies on electronic properties and microstructure of AgBr_1−*x*_Cl*_x_* crystal are required for insight into optimal growth of AgNWs [33,34,39,40,41,42]. With these high aspect ratio (2100) AgNWs, a conductive film was formed with sheet resistance of 48 Ω/sq and transmittance of 95% at 550 nm, which could be utilized as a transparent electrode in optoelectronic applications.

## Figures and Tables

**Table 1:** Parameters of the AgNWs obtained with different additives of NaCl (A_2_), NaBr (B_2_) and NaCl/NaBr (C_1_, C_2_, C_3_).

Sample No.	Molar Ratio of AgNO_3_/NaCl/NaBr	Diameter (nm)	Aspect Ratio	Ag(111)/(200) Ratio	Yield of NWs (%)
A_2_	80/2/0	162.5 ± 21.8	360	4.2	94
B_2_	80/0/2	27.5 ± 7.6	230	3.1	30
C_1_	800/2/1	94.4 ± 11.5	380	4.6	97
C_2_	80/2/1	40.8 ± 6.9	2100	8.8	90
C_3_	16/2/1	29.0 ± 3.8	1400	7.0	85

## Supplementary Materials

The following are available online at http://www.mdpi.com/2079-4991/8/3/161/s1, Figure S1: Digital photographs of reaction flasks and the corresponding distribution statistics of diameter and length for the 30 min growth AgNW samples, Figure S2: EDX analysis of the products for the sample obtained with NaCl/NaBr co-additive, Figure S3: XRD patterns of AgBr_1−*x*_Cl*_x_* crystals and the AgCl/AgBr mixture, Figure S4: SEM images of AgNWs obtained with NaCl additive for different concentrations, Figure S5: SEM images of AgNWs obtained with NaBr additive for different concentrations, Figure S6: EDX analysis of AgBr crystal, Figure S7: SEM images of sample obtained with NaCl/NaBr additive for a Cl/Br molar ratio of 1, Figure S8: UV-vis absorbance spectra and XRD patterns for AgNW samples with NaCl/NaBr additive of different concentrations, Figure S9: Distribution statistics of diameter and length for AgNWs with NaCl/NaBr concentrations of 0.12/0.06 mM and 6.0/3.0 mM, respectively, Figure S10: Digital photographs of AgNW films on glass substrates, Figure S11: Optical microscope images of AgNW films on glass substrates. Table S1: Detailed conditions of AgNW synthesis, Table S2: Ultra-thin AgNWs obtained by other researchers using NaCl/NaBr co-additive.

## Abbreviations

The following abbreviations are used in this manuscript:

AgNWs	Silver nanowires
MTPs	Multiply twinned particles
AgNPs	Silver nanoparticles
Ag*_t_*^+^	Electron traps in the silver halides
ETD	Electron trap distribution
LSPR	Localized surface plasmon resonance
ITO	Indium tin oxide
FWHM	Full width at half maximum
PVP	Poly(vinyl pyrrolidone)
EG	Ethylene glycol
XRD	X-ray diffraction
TEM	Transmission electron microscopy
SEM	Scanning electron microscopy
EDX	Energy dispersive X-ray spectroscopy
UV-vis	Ultraviolet-visible spectra
