# Supplementary material for: Understanding the Effects of NaCl, NaBr and Their Mixtures on Silver Nanowire Nucleation and Growth in Terms of the Distribution of Electron Traps in Silver Halide Crystals
Source: Nanomaterials (Basel). 2018 Mar 14;8(3):161. doi: 10.3390/nano8030161 (PMC5869652; doi:10.3390/nano8030161)
Supplement: Supplementary file 1 [file nanomaterials-08-00161-s001.pdf]

Supplementary Information for

Understanding Effects of NaCl, NaBr and Their Mixtures on Silver Nanowire Nucleation and Growth in Terms of the Distribution of Electron Traps in Silver Halide Crystals

Yunjun Rui[1,2*], Weiliang Zhao[1], Dewei Zhu[2], Hengyu Wang[1], Guangliang Song[3], Mark. T Swihart[2*], Neng Wan[4], Dawei Gu[1], Xiaobing Tang[1], Ying Yang[1] and Tianyou Zhang[1]

[1]Department of Applied Physics, Nanjing Tech University, Nanjing 210009, China

[2]Department of Chemical and Biological Engineering, University at Buffalo (SUNY), Buffalo, New York 14260, United States

[3]College of Chemistry and Molecular Engineering, Nanjing Tech University, Nanjing 210009, China

[4]School of Electronic Science and Engineering, Southeast University, Nanjing, 210096, China

*Corresponding author: yjrui@njtech.edu.cn; swihart@buffalo.edu

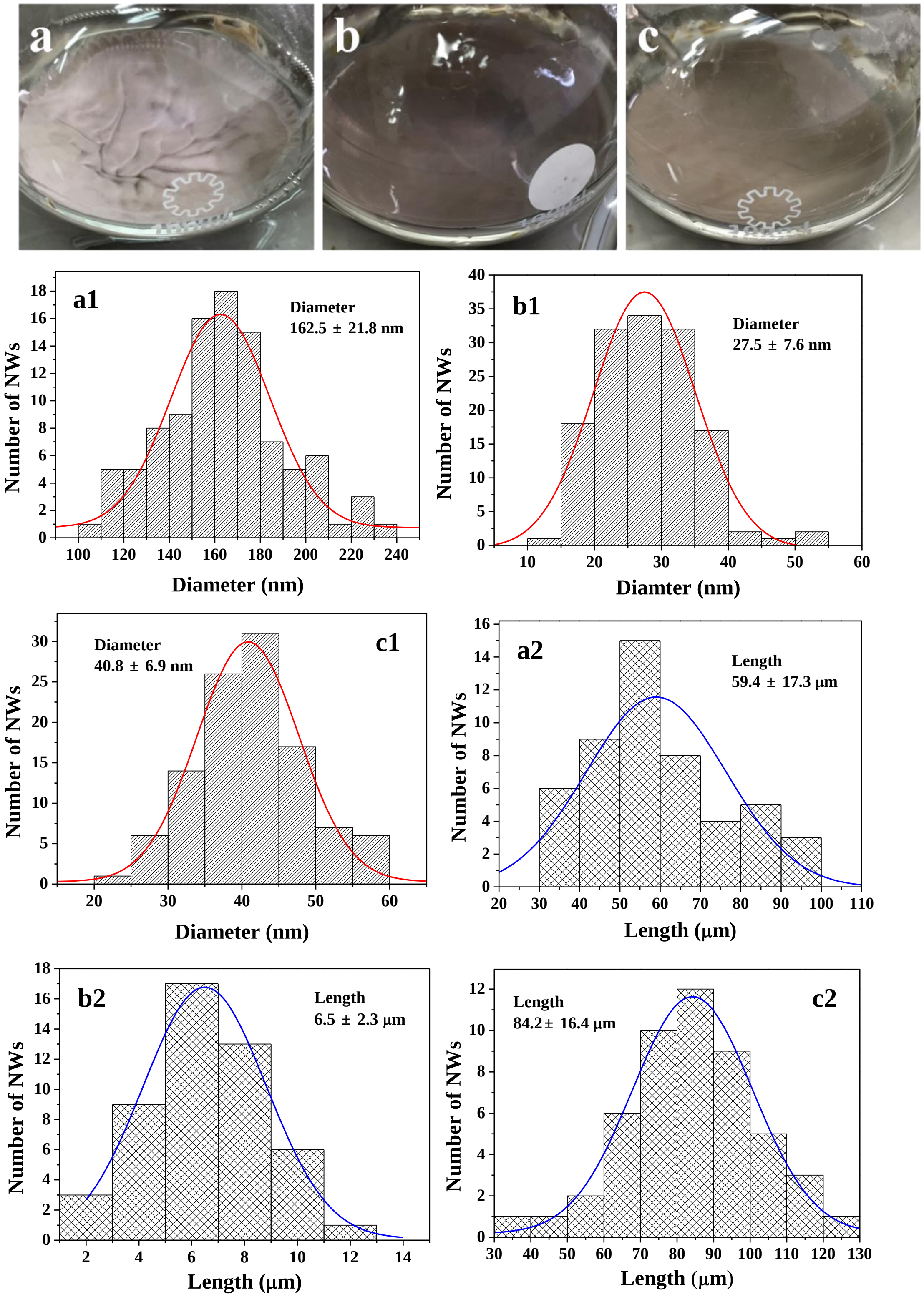

Figure S1. Digital photographs of reaction flasks with densely opalescent (a), slightly gray (b) and silver gray swirls (c), and the corresponding distribution statistics of diameter (a1, b1, c1) and length (a2, b2, c2) for the 30 min growth AgNWs with the additives of NaCl, NaBr and NaCl/NaBr, respectively.

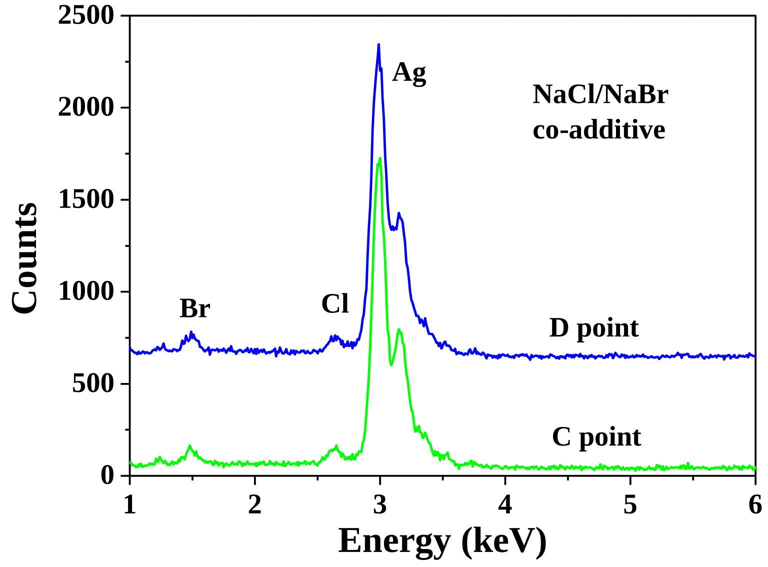

Figure S2. EDX analysis of the products at C and D points for the sample obtained with NaCl/NaBr co-additive shown in Figure 4h in the text.

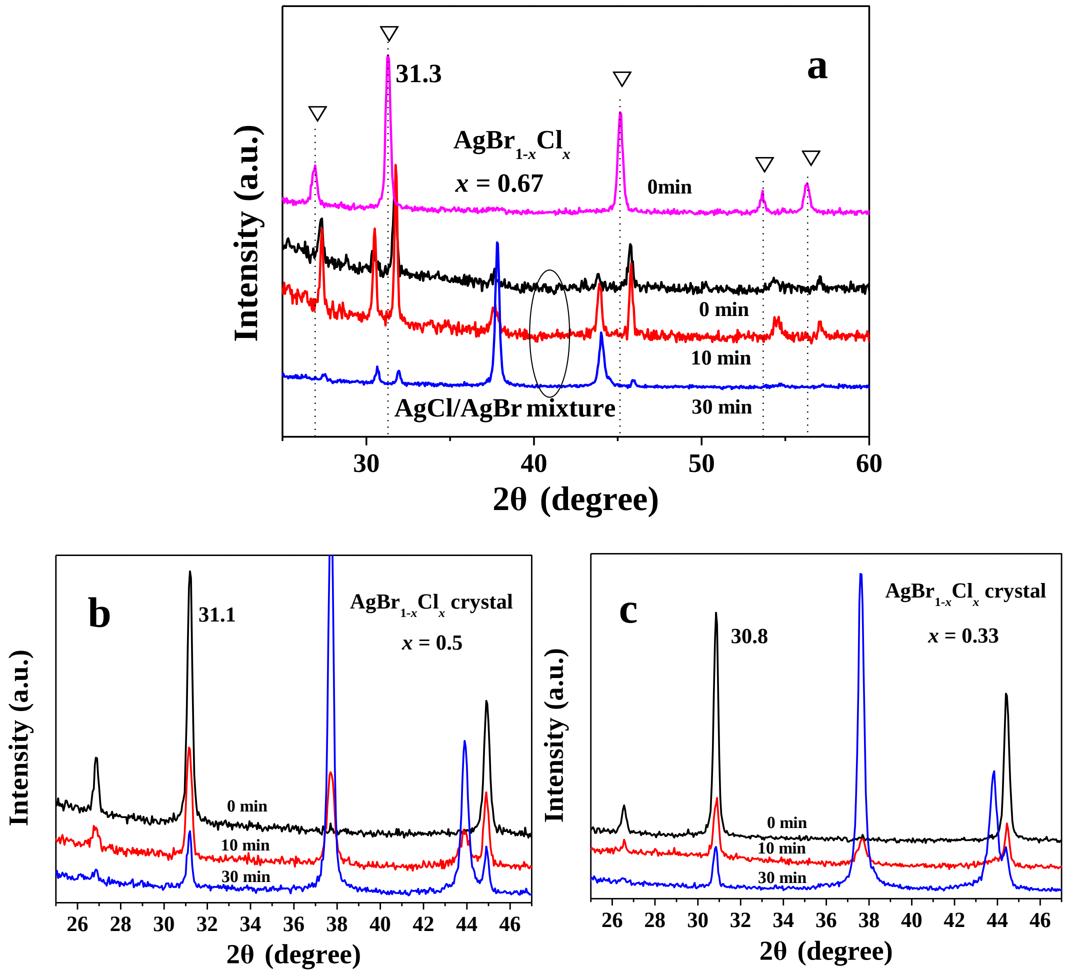

Figure S3. XRD patterns of samples obtained with NaCl/NaBr additive for 0 min growth and the AgCl/AgBr mixture (a). The later was got by simply mixing these two types of AgNWs samples prepared with individual NaCl and NaBr additive. Triangles present the diffraction peaks from $AgBr_{1-x}Cl_x$ crystal, which was quite different with that from AgCl or AgBr crystal. (b) and (c) present the XRD patterns for the evolution of AgNWs samples with NaCl/NaBr concentration of 1.2/1.2 mM and 1.2/2.4 mM, respectively.

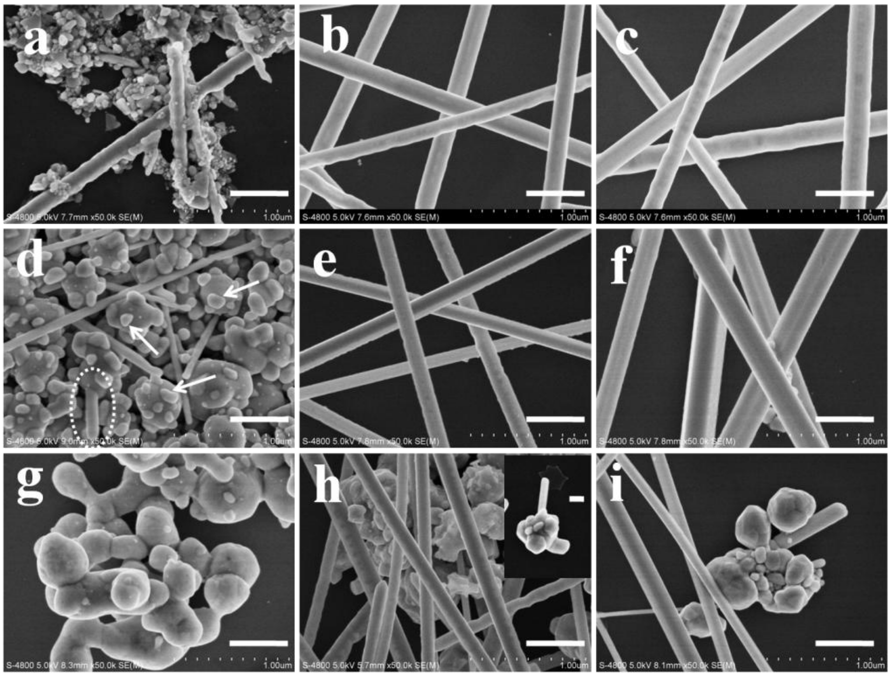

Figure S4. SEM images of AgNWs obtained with NaCl additive for the increasing concentration of 0.12 (a, b, c), 1.2 (d, e, f), 6.0 (g, h, i) at the growth time of 10 (left), 30 (middle) and 60 min (right column). AgNPs (and MTPs) formed on the surface of AgCl crystal were indicated by arrows in (d). The scale bar is 500 nm. The inset in (h) shows one AgNW emanating from the AgBr surface with the scale bar of 100 nm.

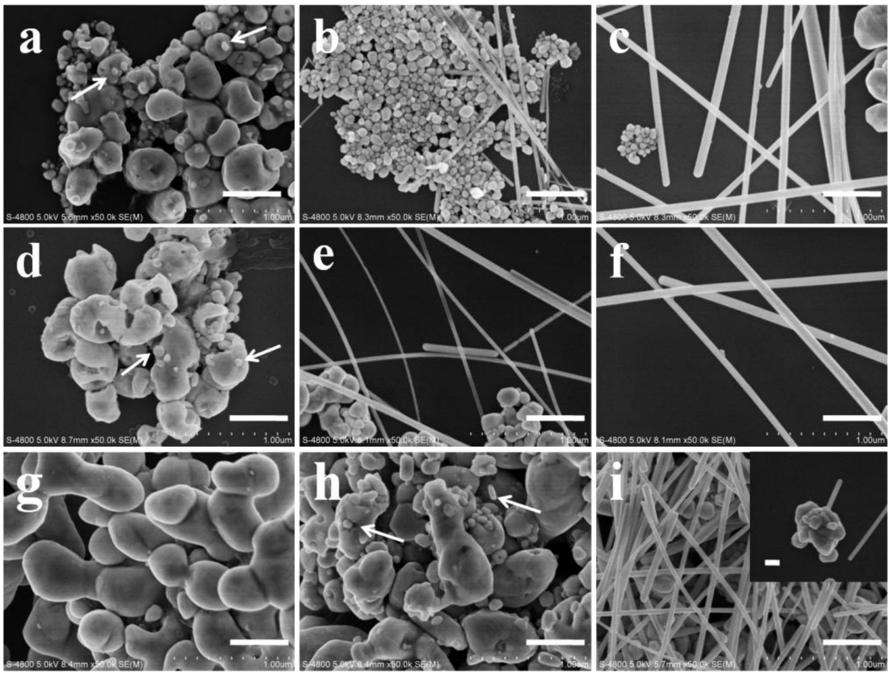

Figure S5. SEM images of AgNWs obtained with NaBr additive for the increasing concentration of 0.12 (a, b, c), 1.2 (d, e, f), 6.0 mM (g, h, i) at the growth time of 10 (left), 30 (middle) and 60 min (right column). AgNPs (and MTPs) formed on the surface of AgBr crystal were indicated by arrows in (a, d, h). The scale bar is 500 nm. The inset in (i) shows one AgNW growing from the AgBr surface with the scale bar of 100 nm.

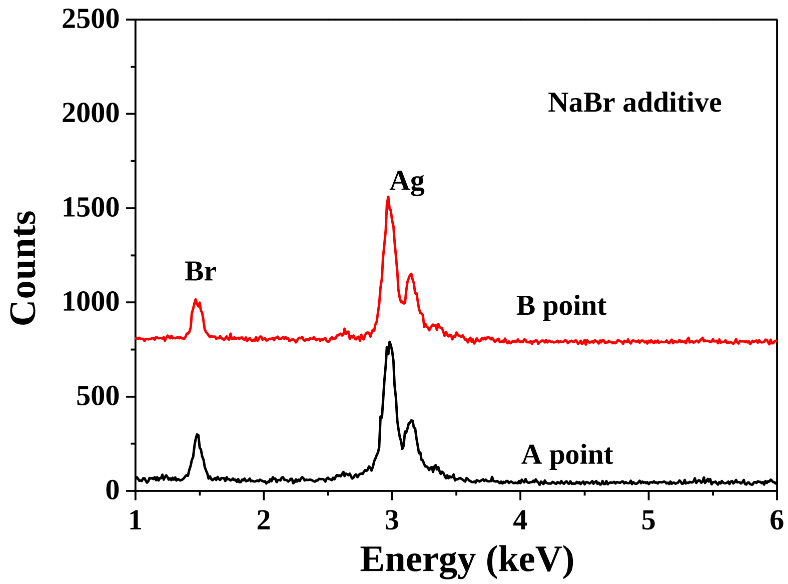

Figure S6. EDX analysis of the products at A and B points for the sample obtained with NaBr additive shown in Figure 4e in the text.

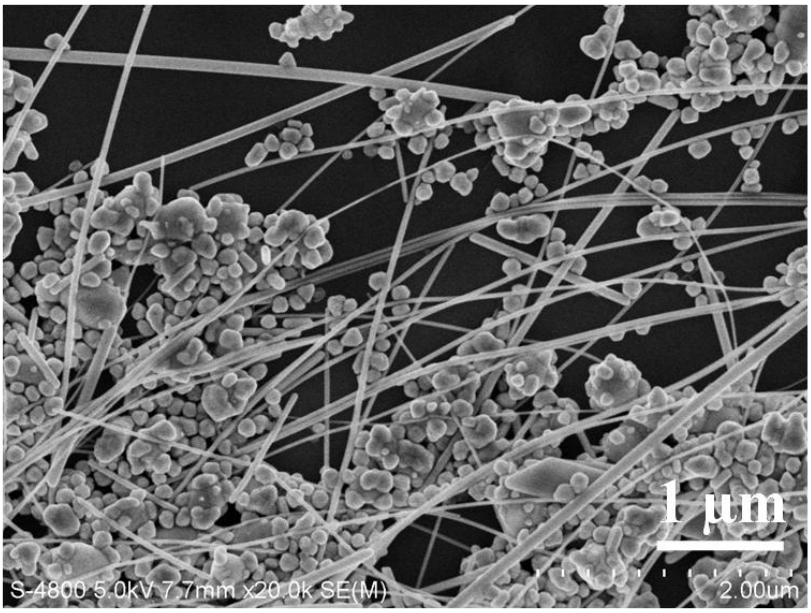

Figure S7. SEM images of sample obtained with NaCl/NaBr additive for a Cl/Br molar ratio of 1.

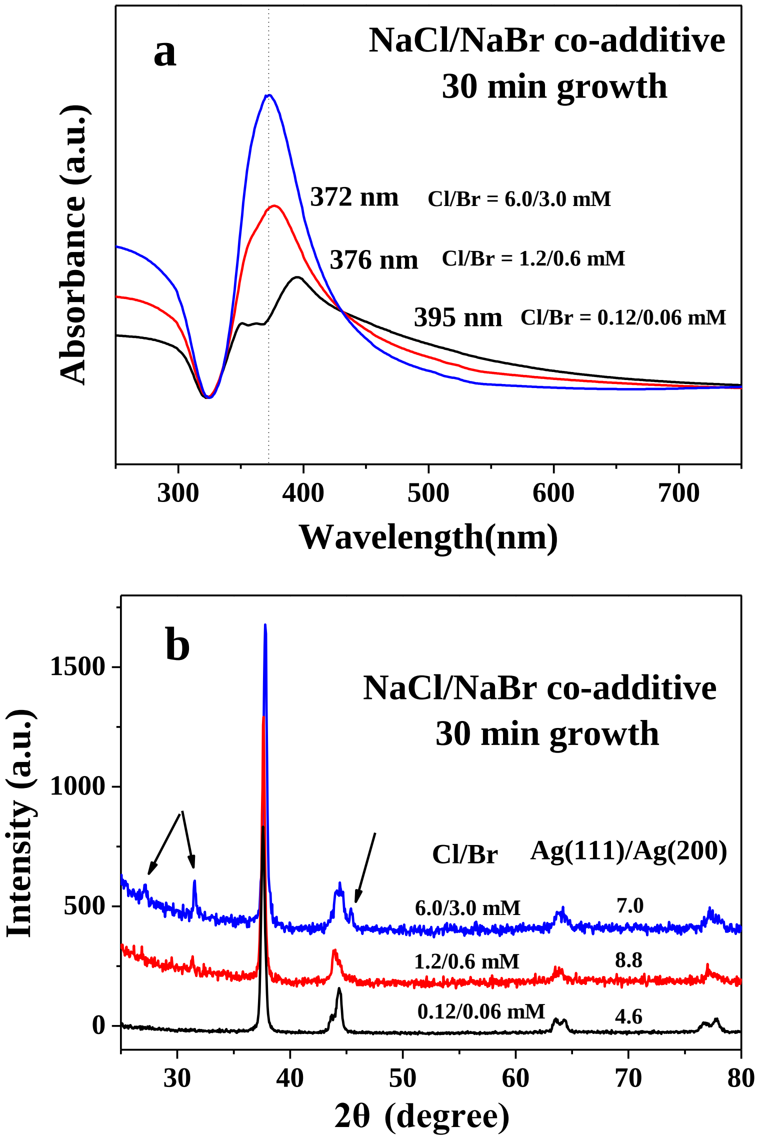

Figure S8. UV-vis absorbance spectra (a) and XRD patterns (b) for 30 min growth samples with NaCl/NaBr additive of different concentrations. The arrows in (b) indicate the $AgBr_{1-x}Cl_x$ crystal remaining in the products.

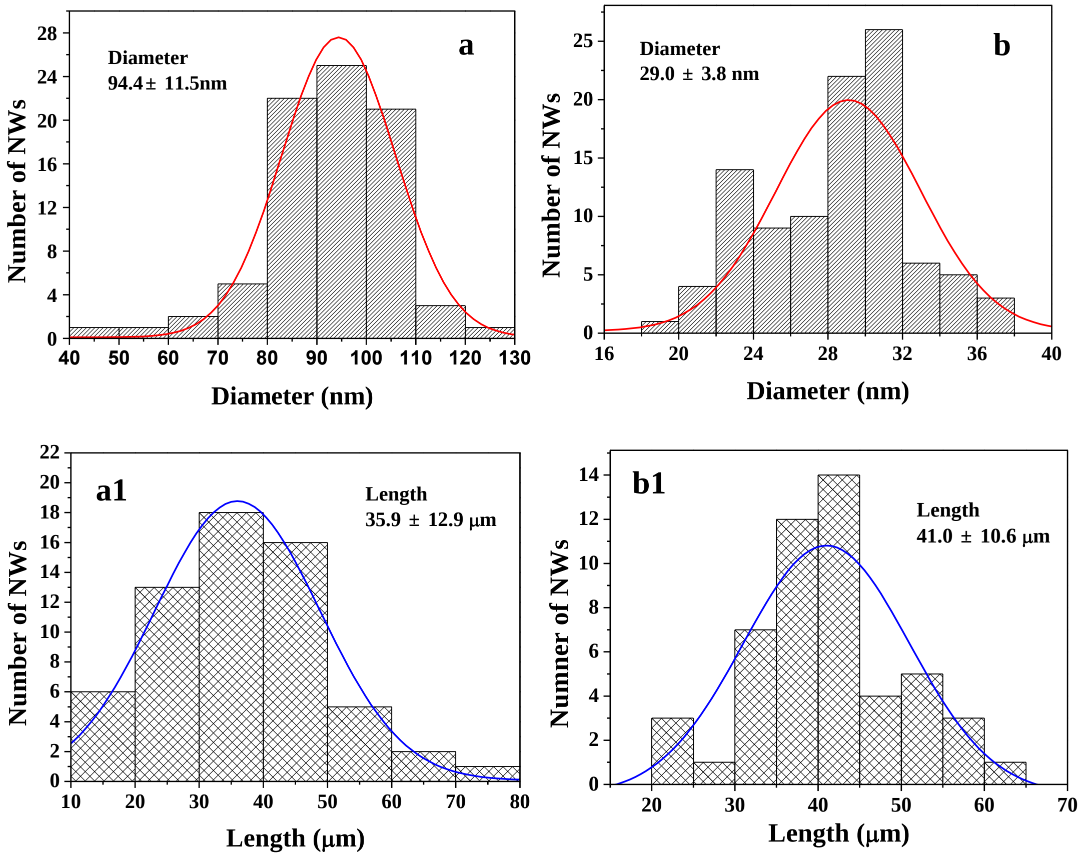

Figure S9. Distribution statistics of diameter (a, b) and length (a1, b1) for the 30 min growth AgNWs with NaCl/NaBr concentrations of 0.12/0.06mM and 6.0/3.0 mM, respectively.

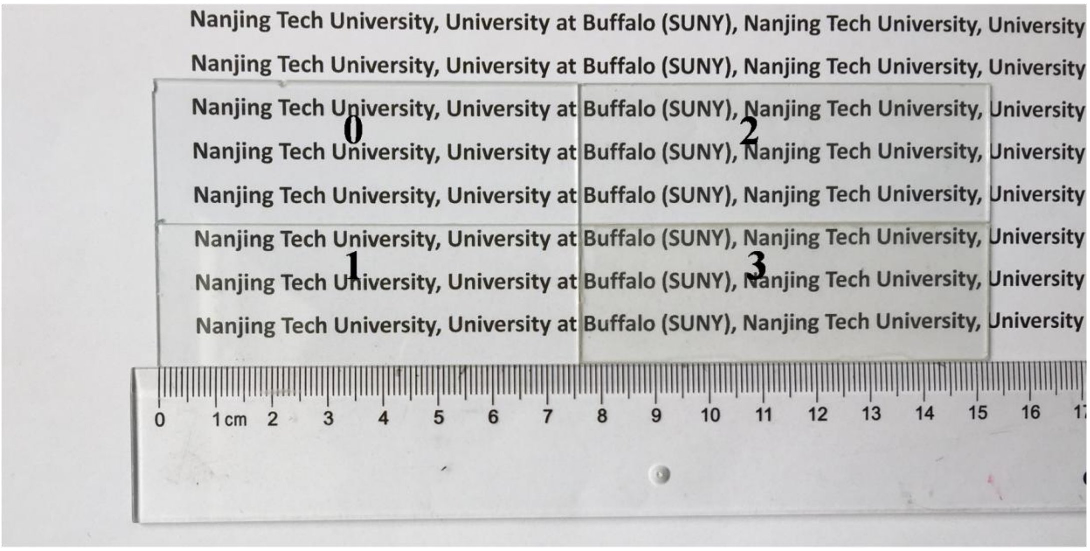

Figure S10. Digital photographs of glass substrate labeled with the number of “0” and the three AgNWs films on glass substrates with transmittance of 98, 95 and 86% labeled with the number of “1”, “2” and “3”, respectively.

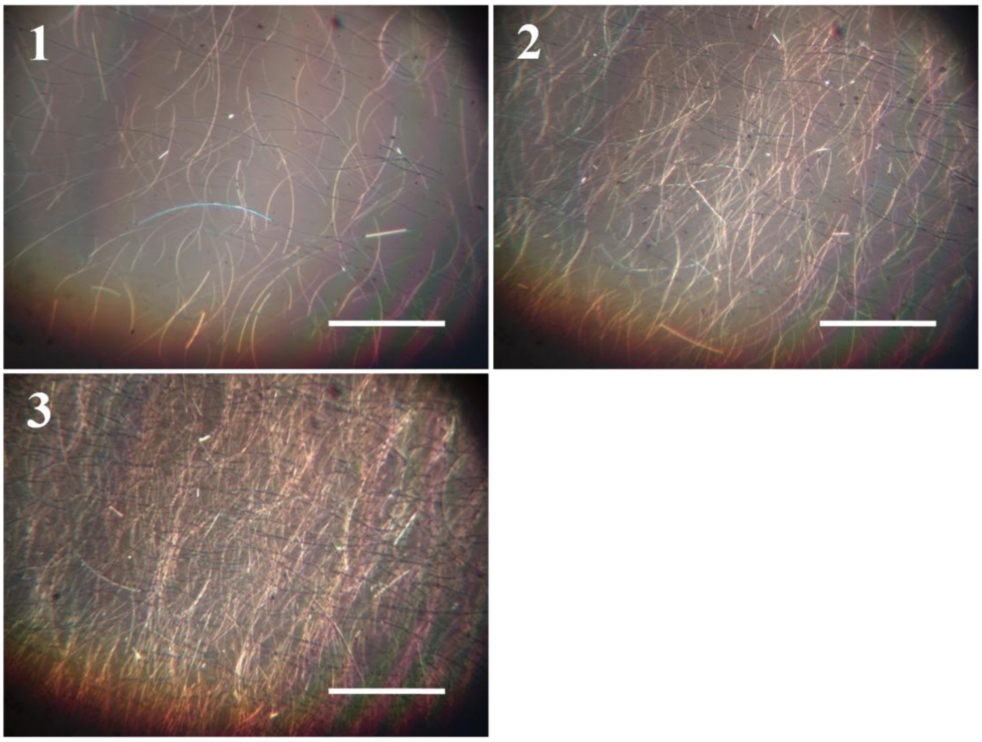

Figure S11. Optical microscope images of the three AgNWs films on glass substrates as described in Figure S10. The scale bar is 50 μm.

Table S1. Detailed conditions of AgNW synthesis and the diameters for 30 min growth samples.

**Sample No.**	**NaCl (mM)**	**NaBr (mM)**	**Diameter (nm)**
A_1	0.12	0	160
A_2	1.2	0	162
A_3	6.0	0	135
B_1	0	0.12	30
B_2	0	1.2	27
B_3	0	6.0	---
C_1	0.12	0.06	94
C_2	1.2	0.6	40
C_3	6.0	3.0	29

Table S2. Ultra-thin AgNWs obtained by other researchers using NaCl/NaBr co-additive with Cl/Br molar ratio of 2 except the last two cases.

**Synthesis atmosphere**	**Molar ratio of $AgNO_3$/NaCl/NaBr**	**Diameter (nm)**	**Aspect ratio**	**AgNW yield (%)**	**References**
N_2	12/2/1	20	2000	57.7	Ref. 1
N_2	13/2/1	20	1500	94.5	Ref. 2
N_2	300/2/1	44	----	----	Ref. 3
N_2	12/2/1	30	----	----	
N_2 high pressure	300/2/1	36	----	----	
N_2 high pressure	12/2/1	16~22	1000	----	
Air	*80/2/1*	*40*	*2100*	*90*	*This work*
Air	*16/2/1*	*29*	*1400*	*85*	*This work*
Air	300/2/1	26	800	----	Ref. 4
Air	16/4/1	30~50	----	50	Ref. 5
Air	60/0/1	Sub-20	1000	85	Ref. 6

References:

1. Li, B.; Ye, S.; Stewart, I.; Alvarez, S.; Wiley, B. Synthesis and purification of silver nanowires to make conducting films with a transmittance of 99%. *Nano Lett.* **2015**, 15, 6722-6726.
2. Jia, D. et al. Synthesis of very thin Ag nanowires with fewer particles by suppressing secondary seeding. *Cryst. Eng. Comm.* **2017**, 19, 148-153.
3. Lee, E.; Kim, Y.; Hwang, D.; Choi, W.; Kim, J. Synthesis and optoelectronic characteristics of 20 nm diameter silver nanowires for highly transparent electrode films. *RSC Adv.* **2016**, 6, 11702.
4. Zhang, K.; Du, Y.; Chen, S. Sub 30 nm silver nanowire synthesized using KBr as co-nucleant through one-pot polyol method for optoelectronic applications. *Org. Electron.* **2015**, 26, 380-385.
5. Hu, L.; Kim, H.; Lee, J.; Peumans, P.; Cui, Y. Scalable coating and properties of transparent, flexible, silver nanowire electrodes. *ACS Nano* **2010**, 4, 2955-2963.
6. Silva, R. et al. Facile synthesis of sub-20 nm silver nanowires through a bromide-mediated polyol method. *ACS Nano* **2016**, 10, 7892-7900.
